# Multi-professional screening instrument for risk of broncho-aspiration in a hospital environment for the elderly population: validity evidence based on response processes

**DOI:** 10.1590/2317-1782/20232022228en

**Published:** 2023-12-22

**Authors:** Paloma Alves Miquilussi, Rosane Sampaio Santos, Giselle Aparecida de Athayde Massi

**Affiliations:** 1 Programa de Pós-graduação em Distúrbios da Comunicação, Universidade Tuiuti do Paraná – UTP - Curitiba (PR), Brasil.

**Keywords:** Deglutition Disorder, Aged, Pneumonia Aspiration, Validation Study, Patient Care Team

## Abstract

**Purpose:**

The objective of this study was to validate the Multiprofessional Screening Instrument for Broncho-aspiration Risk in Hospital Environment, which is aimed at the elderly population, based on response processes.

**Methods:**

Judges applied the instrument to different patients and randomly selected. After the application, the judges were interviewed so that it was possible to verify their impression regarding the relevance of the items about their interpretation of the written content, as well as grammatical and semantic issues. Suggestions for adding alternative questions and answers were considered, as well as proposals for adapting the questions that made up the instrument. Non-verbal reactions, such as facial expressions that suggested doubts or hesitations, by the judges concerning the instrument were also analyzed.

**Results:**

The agreement of the judges concerning each item of the device was calculated by the Content Validity Index (CVI) and by the Intraclass Correlation Coefficient (ICC), and their results showed a high level of agreement. Through the suggestions of the judges, a new version of the Multi-professional Screening Instrument for the Risk of Broncho-aspiration in a Hospital Environment in the Elderly was elaborated.

**Conclusion:**

The results obtained showed that the validity of the Multi-professional Instrument for Screening the Risk of Broncho-aspiration in the Hospital Environment with the elderly population, based on the response processes, was achieved and makes it a promising device to assist professionals in hospital care for the elderly.

## INTRODUCTION

In the process of human ageing, the functionality of swallowing can be compromised due to changes that occur in the neuronal and stomatognathic systems. When these changes are linked to the natural process of ageing, they are referred to as Presbyphagia. However, despite being natural, these changes need to be carefully observed because they can potentially trigger dysphagia, characterised by a deficit in one or more phases of swallowing^(1)^, which can weaken the health of the elderly.

Although it is not necessarily fatal to the individual, over time, dysphagia can cause various damages, such as malnutrition, dehydration, repeated lung infections, and broncho-aspiration^(2)^. Broncho-aspiration, which is the focus of this article, is characterised by the entry of liquid, pasty, and/or solid food, as well as saliva and/or gastric contents, into the lower airways. This health problem can lead to serious conditions, such as aspiration pneumonia, contributing to increased mortality and morbidity rates in the hospitalised population, as well as prolonging hospital stays by an average of 5 to 9 days^(3)^.

In the hospital context, the use of risk screening instruments for broncho-aspiration is a strategy capable of identifying those individuals most likely to have this condition and preventing or mitigating its effects. To this end, the instrument used must have methodological power in its validation processes.

According to the Standard theory, evidence based on the response process seeks data related to the mental aspects involved in carrying out the proposed activity, thus attributing psychological meaning to the application of the items, based on the relationships between their cognitive components^(4)^. Analysing response processes aims to provide evidence of adjustments between the instrument and the detailed nature of the actual response^(5)^.

The Multi-professional Screening Instrument for the Risk of Broncho-aspiration in the Hospital Environment (in Portuguese *Instrumento Multiprofissional de Rastreio para o Risco de Broncoaspiração em Ambiente Hospitalar* (IMRRBAH)) was developed and validated in the first and second stages. The proposal in the first stage was to build an instrument that covered the general population, selecting essential parameters to make up the device, after an extensive literature review on the main indicators related to broncho-aspiration^(6)^, and in the second stage, the IMRRBAH was validated in terms of its form and content.

To continue the process of validating the instrument, the third stage, which is the subject of this study, aims to validate the material based on the response processes. However, in this study, we aimed to restrict the population studied to the elderly, because this population is more susceptible to recurrent hospital admissions, as well as being more fragile due to their inherent functional decline^(7)^.

It should be noted that the validation of the material based on the response processes, resulting from the observation/judgement of the evaluator's behaviour, is necessary to tackle the problem in the design of the questions that make up the instrument, making it possible to structurally modify the tool taking into the account the applicators' observations^(8)^. To this end, the tool must be effectively applied to verify its effectiveness in a real context^(8)^.

This article, which is the third stage in the validation of the IMRRBAH, aims to understand the psychological and cognitive processes of the judges during the application of this instrument.

## METHODS

This is an observational, cross-sectional study with a qualitative and quantitative validation approach. It was carried out in a reference hospital for elderly care. The study was approved by the Research Ethics Committee of the Municipal Health Department of Curitiba under opinion No. 4.992.804.

All the professionals taking part in the research were informed of the study's objectives and signed the Informed Consent Form (ICF). Fifteen professionals were included in the study as applicators of the screening tool, from the following specialties: three geriatricians, three speech therapists, three physiotherapists, three nurses, and three nutritionists. The criterion used to select these professionals was at least two years of experience in caring for hospitalised elderly people.

The 45 participating patients were randomly recruited as follows: every Monday, the first three hospitalised patients who met the inclusion criteria were assigned by the researcher to one of the evaluators. The inclusion criteria for patients were those aged 60 or over, of both sexes, hospitalised for no more than 24 hours, with stable Oxygen Saturation (SpO_2_), Heart Rate (HR), and Respiratory Rate (RR). Patients who agreed to take part in the study also signed an ICF. Concerning exclusion criteria, patients dependent on mechanical ventilation were not included in the study.

Following the recommendations of the Standard Theory concerning evidence based on response processes, after selecting the professionals and signing the ICF, the judges were individually handed the IMRRBAH. On handing the instrument to each of the judges, the researcher carried out standardised oral instruction, according to the steps explained below.

In the first stage, the judge was asked to read all the questions that made up the instrument out loud. The researcher then asked if there were any difficulties in understanding the questions. Also at this stage, the researcher explained that questions 1, 2, 3, 4, 5, 6, and 9 could be answered using data from the patient's medical records. Concerning question 3, if the judges could not find the data in the medical records, they were instructed to calculate the Updated Glasgow Coma Scale (GCS) according to three criteria: eye-opening, verbal response, and motor response.

Concerning questions 7 and 10, the judges were instructed to ask the patient directly. As for question 8, which asks about the patient's oral hygiene, the judges were instructed to carry out an oral inspection to observe the condition of the teeth, as well as the presence or absence of food residue. After all the explanations, the researcher answered any questions that remained, until each judge said they had understood all the instructions.

Following that, each evaluator applied the screening instrument to three different patients and after this stage, the judges were interviewed using a semi-structured script to help the researcher look for evidence of validity based on the response processes.

Issues related to the relevance of the instrument's items were analysed, using the Content Validity Index (CVI) to check the judges' agreement with each item in the instrument^(9)^. The Interclass Correlation Coefficient (ICC) was also calculated, *i.e.*, a coefficient used to estimate the reliability of measures when comparing two or more evaluators^(10)^.

The interpretation of the written content was also analysed, as well as grammatical and semantic issues. This process took into account suggestions for adding alternative answers or questions, as well as suggestions for adapting the questions that made up the instrument. In addition, non-verbal reactions, such as facial expressions, which suggested doubt on the part of the judges concerning the instrument, were analysed^(11)^.

The data obtained was analysed qualitatively and quantitatively. From a qualitative point of view, the responses were analysed based on the judges' descriptions of their general impressions of the instrument, their doubts during its application, and suggestions for changes, additions, or deletions of items. The information was also taken into account as follows: 1) the time taken to apply the instrument; 2) understanding of the psychological and cognitive processes related to the instrument; 3) differences in interpretation. From a quantitative perspective, the responses were evaluated using relative and absolute frequencies. Individual interpretations, the absolute number and percentage of interviewees who did not understand the questions from a semantic point of view, and suggestions for improving the instrument were considered and analysed^(11)^. After analysing the answers, the relevant changes were made, culminating in the development of a new version of the IMRRBH, aimed at the elderly population.

## RESULTS

The professionals taking part in the study are on average 34 years old, with a minimum age of 25 and a maximum of 53. The profile of the participating professionals is described in Table 1.

**Table 1:** Distribution of data regarding the profile of the study participants

Variables	n	%
Age		
25 to 39 years old	12	80.0
40 years or older	3	20.0
Academic level		
Postgraduate degree	12	80.0
Master’s degree	3	20.0
Practice area		
Only clinical practice	6	40.0
Clinical practice and teaching	9	60.0
Length of clinical practice		
Between 2 and 5 years	5	33.3
Between 5 and 10 years	3	20.0
10 years or more	7	46.7

Caption: n = number

Source: Authors

### Verbal responses

The CVI value varied between 0.93 and 1.0. The ideal value to consider a high level of agreement is 0.90^(9)^. These data are described in Table 2.

**Table 2:** Verbal responses

Questions	CVI	Professionals considered the issue relevant or very relevant	Professionals considered the question to be clearly written	Professionals kept the wording of the questions without any suggestions for changes	Professionals kept the binomial yes/no answer	Professionals kept the questions with no suggested additions
		n (%)	(%)	(%)	(%)	(%)
**1)** Patient aged ≥ 60 years?	1	15 (100)	80	73.3	93.3	100
() yes () no						
**2)** Do you have a history of previous illnesses (neurological, respiratory, oesophageal, gastric, cx of head and neck)?	1	15 (100)	86.6	80	93.3	86.6
() yes () no						
**3)** Is the patient with a GCS score of < 13?	1	15 (100)	73.3	66.6	100	93.3
() yes () no						
**4)** Did you require Orotracheal Intubation?	1	15 (100)	73.3	60	86.6	86.6
() yes () no						
**5)** Orotracheal intubation time ≥ 24 hours?	0.93	14 (93.3)	80	46.6	93.3	86.6
() yes () no						
**6)** Do you use tracheostomy?	1	15 (100)	100	86.6	100	73.3
() yes () no						
**7)** Does the patient experience dyspnea?	1	15 (100)	100	86.6	86.6	66.6
() yes () no						
**8)** Does the patient have poor oral hygiene?	0.93	14 (93.3)	73.3	53.3	93.3	60
() yes () no						
**9)** Does the patient use an alternative feeding route (NES, GTT, Jejunostomy)?	1	15 (100)	100	86.6	86.6	73.3
() yes () no						
**10)** Does the patient have a cough/gasp while eating or with saliva?	1	15 (100)	100	100	93.3	66.6
() yes () no						

Caption: CVI = Content Validity Index; NES = Nasoenteral tube; GTT = Gastrostomy; GCS = Glasgow Coma Scale

Source: Authors

The ICC ranged from 0.88 to 0.97, indicating an excellent level of agreement^(9)^. Thus, the high value of the ICC suggests that the variability between the evaluators' responses was low, indicating a positive result in the agreement analysis. The calculations of these results are described in Table 3.

**Table 3:** Intraclass Correlation Coefficient - ICC

**ITEM**	**ICC**	Confidence interval - 95%	
		Lower limit	Upper limit
1	0.952	0.886	0.999
2	0.961	0.883	0.999
3	0.945	0.836	0.999
4	0.925	0.878	0.999
5	0.891	0.868	0.999
6	0.906	0.882	0.999
7	0.962	0.886	0.999
8	0.884	0.865	0.999
9	0.969	0.908	0.999
10	0.973	0.921	0.999
**Total**	**0.931**	**0.900**	**0.996**

Source: Authors

### Non-verbal responses

In addition to the questionnaire answered by the participating professionals, non-verbal responses were verified, based on four gestures expressed by them: hesitation, frowning, tilting the head, and directing the hand to the mouth. The data obtained is shown in Table 4, which indicates that there was at least one type of gesture indicating a non-verbal response in each item. In items 3 and 8, there was more frequent hesitation - in which at least three (20.0%) of the participants expressed this gesture - as well as frowning, tilting the head, and directing the hand to the mouth, in the case of item 8. On the other hand, in item 9, only one (6.7%) of the participants exclusively expressed the gesture of frowning as a non-verbal response to the item.

**Table 4:** Frequency distribution of non-verbal responses by each item

**ITEMS**	**Hesitation**		**Frowning**		**Tilting head**		**Hand to mouth**	
	n	%	n	%	n	%	n	%
1	2	13.3	1	6.7	-	-	-	-
2	1	6.7	2	13.3	-	-	1	6.7
**3**	**4**	**26.7**	**2**	**13.3**	**1**	**6.7**	**-**	**-**
4	2	13.3	2	13.3	1	6.7	-	-
5	2	13.3	-	-	1	6.7	-	-
6	1	6.7	-	-	1	6.7	-	-
7	2	13.3	-	-	-	-	1	6.7
**8**	**3**	**20.0**	**1**	**6.7**	**2**	**13.3**	**2**	**13.3**
**9**	**-**	**-**	**1**	**6.7**	**-**	**-**	**-**	**-**
10	1	6.7	-	-	-	-	1	6.7

Caption: n = number

Source: Authors

Concerning general suggestions for the instrument, in question 2, one judge said that he needed to explain the question to the patient and another judge said that he needed to give examples of diseases to which he was referring. In question 3, one judge suggested adding the RASS - Richmond Agitation Sedation Scale, if the instrument could be adapted for use in Intensive Care Units (ICUs). Three judges said that they were unfamiliar with the GCS, so they were unfamiliar with how to apply it. Also about this question, one judge mentioned that the GCS is validated for assessing patients who have suffered trauma, suggesting that a more appropriate scale should be used to screen for the risk of bronchoaspiration without, however, indicating another option.

In question 4, one judge said that he had to explain the term “Orotracheal Intubation” to the patient. In question 7, one judge suggested that the applying professional could describe whether they noticed signs of respiratory discomfort during the screening. Another judge said he needed to explain the meaning of the word “dyspnea”. Finally, in question 10, one judge suggested that the professional applicator could describe issues related to the efficiency of the patient's cough. General suggestions for the instrument included patient identification, the date the screening instrument was applied, and the patient's medical record number.

After analysing the judges' suggestions, changes were made to the instrument, taking into account relevant data to make it easier to use. These adjustments are shown in Chart 1.

**Chart 1:** Adjustments made after analysing the interviews and consensus between the authors

*Questions from the first version of the Multiprofessional Screening Instrument for Bronchoaspiration Risk in the Hospital Environment*	Adaptations for the second version of the Instrument
1. Is the patient ≥60 years old?	Question format removed and answers to this question adapted. Replacement of the symbol with its full spelling, as it could be noted that this would cause less confusion for individuals unfamiliar with the symbolism.
2. Do you have a history of previous illness? (Neurological, Respiratory, Oesophageal, Gastric, cx of Head and Neck)	Inclusion of the word “current”, thus comprising not only the history of previous illnesses and modification of the suggestions of illnesses to also encompass “head and neck illnesses”.
3. Is the patient with a GCS score of < 13?	Replacing the symbol by writing it in full.
4. Did you need Orotracheal Intubation?	Inclusion of the term “in this hospitalisation? “, as this scenario would have an impact on the patient's current condition.
5. Orotracheal intubation time (OIT) ≥ 24 h?	Replacement of the symbol with its full spelling. Inclusion of the term “in this hospitalisation” and inclusion of the answer option “not applicable”.
6. Do you use a tracheostomy?	No change.
7. Does the patient experience dyspnea?	Inclusion of the answer option “not applicable”, considering that only the patient can answer this question and some patients are unable to due to their clinical condition.
8. Does the patient have poor oral hygiene?	Replacement of the term inadequate with adequate.
9. Does the patient use an alternative feeding route (NES, GTT, Jejunostomy)?	Addition of the term “as the only route”, since some patients only need this route as a nutritional supplement. Inclusion of the alternative parenteral route (PN), as it is a feeding route present in the hospital environment.
10. Does the patient cough/gasp while eating or with saliva?	Inclusion of the answer option “not applicable”, considering that only the patient can answer this question and some patients are unable to due to their current clinical condition or underlying illnesses.

Caption: NES = Nasoenteral tube; GTT = Gastrostomy; PN = Parenteral Nutrition; GCS = Glasgow Coma Scale; OIT = Orotracheal Intubation Time

Source: Authors

Chart 2 shows the questions from the first version of the instrument, the judges' suggestions, and what was considered for the second version of the instrument.

**Chart 2:** Comparative data between the first and second versions of the instrument

**Items that made up the first version of the Instrument**	**Suggested modifications**	**Items considered for the second version of the instrument**
1) Is the patient ≥ 60 years old? *Answer:* () No () Yes	*Question:* a) Age: (b) Is the patient older than or equal to 60 years? Answer: a) () 60-74 years, b) () over 75 years old	1) Age: () between 60 and 74 years old () over 75
2) Do you have a history of previous illnesses (neurological, respiratory, oesophageal, gastric, cx of head and neck)? *Answer:* () No () Yes	*Question:* a) History of previous illness: (Neurological, Respiratory, Oesophageal, Gastric, cx of Head and Neck). b) History of current or previous illness: (Neurological, Respiratory, Oesophageal, Gastric, cx of Head and Neck or neoplasm); c) Any history of previous illness? (Add: stroke, Parkinson's, dementia) Answer: (a) () present, () absent b) What comorbidities?	2) Do you have a history of current or previous illness (neurological, gastric, respiratory, or head and neck disease)? *Answer:* () No () Yes
3) Is the patient with a GCS score of < 13? *Answer:* () No () Yes	*Question:* a) Is the patient with a GCS score greater than 13? b) GCS less than 13: c) Is the patient with a GCS score less than 13? d) Is this the patient's baseline waking state?	3) Is the patient with a GCS score of less than 13? *Answer:* () No () Yes
4) Did you require Orotracheal Intubation (OI)? *Answer:* () No () Yes	*Question:* a) Did you require OI during this hospitalisation? b) OI: c) How many days? d) How long? e) During this hospitalisation? f) Did you stay in the ICU?	4) Did you require OI during this hospitalisation? *Answer:* () No () Yes
5) Orotracheal intubation time (OIT) ≥ 24 h? *Answer:* () No () Yes	*Question:* a) OIT greater than or equal to 24 h? b) OIT ≥ 24 h: c) Time of OIT ≥ 24 h, in this hospitalisation? d) How many days? e) Cause of OIT? *Answer:* a) () Not applicable	5) OIT greater than or equal to 24 hours in this hospitalisation? *Answer:* () No () Not applicable () Yes
6) Do you use a tracheostomy? *Answer:* () No () Yes	*Question:* a) Tracheostomy: b) Did you use a tracheostomy during this hospitalisation? c) Has extubation failed? d) How long have you been using it? e) Since this hospitalisation? f) Have you used it before? *Answer:* a) () present () absent	6) Do you use a tracheostomy? *Answer:* () No () Yes
7) Does the patient experience dyspnea? *Answer:* () No () Yes	*Question:* a) Dyspnea: b) Is the patient short of breath? c) Do you use O_2_ support? d) At what times of the day? e) When is it most intense? f) How often? g) Was this the reason for hospitalisation? *Answer:* a) () not applicable	7) Does the patient feel “short of breath”? *Answer:* () No () Not applicable () Yes
8) Does the patient have poor oral hygiene? *Answer:* () No () Yes	*Question:* a) Does the patient have adequate oral hygiene? b) Oral hygiene: c) Does the patient have poor oral hygiene? d) Do you use dentures? e) Do you perform oral hygiene alone? f) How often do you perform oral hygiene? g) Why is oral hygiene poor? *Answer:* a) () Partially adequate	8) Does the patient have poor oral hygiene? *Answer:* () Yes () No
9) Does the patient use an alternative feeding route (NES, GTT, Jejunostomy)? *Answer:* () No () Yes	*Question:* a) Alternative feeding route: (NES, GTT, Jejunostomy) b) Does the patient use an alternative feeding route as the sole route? c) Does the patient use an alternative feeding route? (NES, GTT, Jejunostomy, PN) d) How long have you been using an alternative feeding route? e) Have you used them before? f) Why are you using the device?	9) Does the patient use a feeding tube as the only route? (NES, GTT, Jejunostomy, PN). *Answer:* () No () Yes
10) Does the patient have a cough/gasp while eating or with saliva? *Answer:* () No () Yes	*Question:* a) Positioning in bed: _______ b) What type of consistency does the patient eat? c) With what foods? d) How long have they been having these episodes? e) When did these symptoms start? f) How often? g) What is the cause of the coughing/sniffing? *Answer:* a) () not applicable	10) Does the patient cough/gasp while eating or with saliva? () No () Not applicable () Yes

Caption: NES = Nasoenteral tube; GTT = Gastrostomy; PN = Parenteral Nutrition; ICU = Intensive Care Unit; GCS = Glasgow Coma Scale; OIT = Orotracheal Intubation Time; OI = Orotracheal Intubation

Source: Authors

Thereupon, when analysing the results obtained during the interviews, the second version of the IMRRBAH for the elderly population was drawn up, including a written instruction guide to make it easier for professionals to use.

## DISCUSSION

This research aimed to improve the IMRRBAH for the elderly population, which was developed for application by health professionals involved in direct patient care. As a result, it was decided to find validation evidence based on the response processes, through directed interviews and analysis of the psychological processes involved.

Concerning the profile of the judges, it can be seen that all of them had some specialisation and there was a predominance of professionals who, in addition to their clinical work, which most of them had been doing for more than 10 years, also taught. In this sense, the literature points out that the skills developed by specialised professionals play an important role in the permanent development of health professionals, capable of modifying their practice through new models of care, contributing effectively to the institutions in which they work as well as benefiting this population^(12)^.

The CVI was used to quantitatively analyse the verbal responses, as it is a method widely used in the health field to measure the judges' agreement with each item that makes up a given instrument individually using the Likert scale^(13)^.

According to most of the judges, the item wording was clear. In addition, the majority said they would keep the way the questions and answers were presented, which is in line with the scientific literature, which states that validated instruments should be made up of coherent and relevant items on the subject they are intended to assess, avoiding doubts on the part of the professional^(14)^.

With respect to the non-verbal responses obtained, we would highlight those found in item 8 of the instrument, referring to the patient's oral hygiene, as it was the question that caused the most strangeness on the part of the judges, which is why the suggestion of changing the term “inadequate” to “adequate” was considered.

The other suggestions made by the judges and considered pertinent were chosen to make up the new version of the instrument, as well as the instruction guide for its application.

The search for other indications of validity is necessary, especially with regard to validating the scores assigned in the previous stages, to obtain more consistent and concrete data for screening on when to call a speech therapist to carry out a clinical assessment of swallowing. A recent search revealed that the device, which is the subject of this study, is still the only one in the scientific literature that aims to track the risk of bronchoaspiration, a fact that demonstrates the relevance of continuing this research. The materials found in the literature aim to track the risk of dysphagia^(15,16)^, which is a risk predictor for bronchoaspiration, but should not be the only one to be considered. Other studies also point to the importance of preventing bronchoaspiration and suggest prevention protocols for this condition^(17,18)^.

Regarding the study's limitations, it should be emphasised that it was carried out in a hospital that is a reference in the care of the elderly, which restricted the sample population.

## CONCLUSION

The results obtained in the third stage of this research showed, through data from the judges' psychological and cognitive processes, that the validity of the IMRRBAH with the elderly population based on the response processes was achieved.
