# Supplementary material for: Multi-professional screening instrument for risk of broncho-aspiration in a hospital environment for the elderly population: validity evidence based on response processes
Source: Codas. 2023 Dec 22;36(1):e20220228. doi: 10.1590/2317-1782/20232022228en (PMC10750859; doi:10.1590/2317-1782/20232022228en)
Supplement: Translation

# Translation: Instrumento multiprofissional de rastreio para risco de broncoaspiração em ambiente hospitalar à população idosa: evidências de validade baseada nos processos de resposta

**DOI:** 10.1590/2317-1782/20232022228pt

**Authors:** Paloma Alves Miquilussi, Rosane Sampaio Santos, Giselle Aparecida de Athayde Massi

**Affiliations:** 1 Programa de Pós-graduação em Distúrbios da Comunicação, Universidade Tuiuti do Paraná – UTP - Curitiba (PR), Brasil.

**Keywords:** Transtornos de Deglutição, Idoso, Pneumonia Aspirativa, Estudo de Validação, Equipe de Assistência ao Paciente

## Abstract

**Objetivo:**

O objetivo deste estudo foi validar, com base nos processos de resposta, o Instrumento Multiprofissional de Rastreio para o Risco de Broncoaspiração em Ambiente Hospitalar, direcionado à população idosa.

**Método:**

O instrumento foi aplicado por juízes em pacientes distintos e selecionados de forma aleatória. Após a aplicação, os juízes foram entrevistados para que fosse possível verificar a impressão deles quanto à relevância dos itens e quanto a interpretação sobre o conteúdo escrito, bem como a questões gramaticais e semânticas. Foram consideradas sugestões de acréscimo de alternativas de perguntas e de respostas, além de propostas de adequação de questões que compunham o instrumento. As reações não verbais, tais como expressões faciais que sugeriram dúvidas ou hesitações, por parte dos juízes, em relação ao instrumento, também foram analisadas.

**Resultados:**

A concordância dos juízes em relação a cada item do dispositivo foi calculada pelo Índice de Validade de Conteúdo (IVC), e pelo Coeficiente de Correlação Intraclasse (CCI), sendo que seus resultados demonstraram alto nível de concordância. Através das sugestões dos juízes, elaborou-se uma nova versão do Instrumento Multiprofissional de Rastreio para o Risco de Broncoaspiração em Ambiente Hospitalar à população idosa.

**Conclusão:**

Os resultados obtidos demonstraram que a validade do Instrumento Multiprofissional para Rastreio do Risco de Broncoaspiração em Ambiente Hospitalar junto à população idosa, baseada nos processos de respostas, foi alcançada.

## INTRODUÇÃO

No processo de envelhecimento humano, a funcionalidade da deglutição pode ficar comprometida devido a mudanças que ocorrem nos sistemas neuronal e estomatognático. O conjunto dessas mudanças, quando se vincula ao processo natural do envelhecimento, é denominado de Presbifagia. Porém, apesar de naturais, tais modificações precisam ser observadas com atenção pelo fato de se apresentarem potencialmente desencadeadoras de um quadro de Disfagia, caracterizada pelo déficit de uma ou mais fases da deglutição^(1)^, capaz de fragilizar a saúde do idoso.

Embora, não seja necessariamente fatal ao indivíduo, ao longo do tempo, a Disfagia pode acarretar vários danos, tais como desnutrição, desidratação, infecções pulmonares de repetição e broncoaspiração^(2)^. A broncoaspiração, que é ponto central deste artigo, é caracterizada pela entrada de alimentos líquidos, pastosos e/ou sólidos, além de saliva e/ou conteúdo gástrico, nas vias aéreas inferiores. Tal condição pode acarretar quadros graves, como a pneumonia aspirativa, contribuindo para aumentar as taxas de mortalidade e morbidade da população, em situação de internamento hospitalar, bem como para prolongar o período de internamento de 5 a 9 dias, em média^(3)^.

No contexto hospitalar, a aplicação de instrumentos de rastreio de risco para broncoaspiração configura-se como uma estratégia capaz de identificar os indivíduos com maior probabilidade de apresentar tal condição, impedindo ou atenuando os seus efeitos. Para tanto, é crucial que o instrumento utilizado apresente potência metodológica em seus processos de validação.

De acordo com a teoria dos Standards, as evidências baseadas no processo de resposta buscam dados relacionados aos aspectos mentais envolvidos na execução da atividade proposta, atribuindo significado psicológico para a aplicação dos itens, a partir das relações entre seus componentes cognitivos^(4)^. A análise dos processos de resposta visa fornecer evidências sobre ajustes entre o instrumento e a natureza detalhada da resposta real^(5)^.

O Instrumento Multiprofissional de Rastreio para o Risco de Broncoaspiração em Ambiente Hospitalar (IMRRBAH) foi desenvolvido e validado, em primeira e segunda etapas, sendo que a proposta da primeira etapa foi construir um instrumento que contemplasse a população em geral, selecionando parâmetros essenciais para compor o dispositivo, após extensa revisão bibliográfica acerca dos principais indicativos relacionados à broncoaspiração^(6)^ e na segunda etapa foi elaborada a validação do IMRRBAH quanto a forma e conteúdo do instrumento.

Para dar seguimento ao processo de validação do instrumento, a terceira etapa, que se constitui como objeto do presente estudo, é a de validação do material, a partir dos processos de resposta. No entanto, nesta pesquisa optou-se por restringir a população estudada, elegendo o idoso, pelo fato de que esta população é mais suscetível a internações hospitalares recorrentes, além de apresentar maior fragilidade relacionada ao seu inerente declínio funcional^(7)^.

Cabe ressaltar que a validação do material a partir dos processos de resposta, resultante da observação/julgamento sobre o comportamento do avaliador, é necessária para resolver falhas na elaboração das questões que compõem o instrumento, possibilitando a modificação estrutural da ferramenta diante das observações dos aplicadores^(8)^. Para tanto, o instrumento deve ser efetivamente aplicado a fim de que seja verificada a sua eficácia, em um contexto real^(8)^.

Assim, o objetivo deste artigo, que constitui a terceira etapa de validação do IMRRBAH é compreender os processos psicológicos e cognitivos dos juízes, durante a aplicação de tal instrumento

## MÉTODO

Esta pesquisa configura-se como um estudo observacional, do tipo transversal, de abordagem qualitativa e quantitativa do tipo validação. Foi realizada em um Hospital referência no atendimento ao idoso. O estudo foi aprovado pelo Comitê de Ética em Pesquisas da Secretaria Municipal da Saúde de Curitiba sob o parecer de Nº 4.992.804.

Todos os profissionais, participantes da pesquisa, foram informados quanto aos objetivos do estudo e assinaram o Termo de Consentimento Livre e Esclarecido (TCLE). Foram incluídos na pesquisa, como aplicadores do instrumento de rastreio, 15 profissionais, considerando as seguintes especialidades: três Médicos Geriatras, três Fonoaudiólogos, três Fisioterapeutas, três Enfermeiros e três Nutricionistas. O critério utilizado para a seleção destes profissionais foi a experiência de atendimento ao idoso hospitalizado de, no mínimo, dois anos.

Já, os 45 pacientes participantes, foram recrutados aleatoriamente, da seguinte forma: todas as segundas-feiras, os três primeiros pacientes internados, que atendiam aos critérios de inclusão, foram direcionados pela pesquisadora a um dos avaliadores. Sobre os critérios de inclusão dos pacientes, foram convidados aqueles com idade igual ou acima de 60 anos, de ambos os sexos, internados há, no máximo, 24 horas, que apresentavam Saturação de Oxigênio (SpO_2_), Frequência Cardíaca (FC) e Frequência Respiratória (FR) estáveis. Os pacientes que aceitaram participar do estudo, também assinaram o TCLE. No que se refere aos critérios de exclusão, não fizeram parte da pesquisa pacientes dependentes de ventilação mecânica.

Seguindo as recomendações da teoria dos Standards em relação às evidências baseadas nos processos de respostas, após a seleção dos profissionais e assinatura do TCLE, os juízes receberam individualmente, em mãos, o IMRRBAH. Ao entregar tal instrumento para cada um dos juízes, a pesquisadora realizou instrução oral padronizada, de acordo com as etapas explicitadas na sequência.

Na primeira etapa, foi solicitado que o juiz lesse todas as questões que compunham o instrumento, em voz alta. Em seguida, a pesquisadora perguntou se havia alguma dificuldade para entender tais questões. Ainda nessa etapa, a pesquisadora explicitou que as questões 1, 2, 3, 4, 5, 6, e 9 poderiam ser respondidas por meio de dados que estavam no prontuário do paciente. Em relação à questão 3, caso os juízes não encontrassem o dado no prontuário, eles eram orientados a calcular a Escala de Coma de Glasgow Atualizada, de acordo com três critérios: abertura ocular, resposta verbal e resposta motora.

No que se refere às questões 7 e 10, os juízes foram orientados a fazer as perguntas diretamente ao paciente. Já, a questão 8, que busca saber sobre a higiene oral do paciente, os juízes foram orientados a realizar inspeção oral, a fim de observar a condição dentária, bem como presença, ou não, de resíduo alimentar. Após todas as explicações, a pesquisadora sanou dúvidas que ainda persistiram, até que cada juiz afirmasse ter entendido todas as orientações.

Em seguida, cada avaliador aplicou o instrumento de rastreio em três pacientes distintos e após esta etapa, os juízes foram entrevistados com auxílio de roteiro semiestruturado, a fim de auxiliar a pesquisadora a buscar evidências de validade, baseadas nos processos de resposta.

Foram analisadas questões relacionadas à relevância dos itens do instrumento, para isso utilizou-se o cálculo do Índice de Validade de Conteúdo (IVC), a fim de verificar a concordância dos juízes em relação a cada item do instrumento^(9)^. Calculou-se ainda o Coeficiente de Correlação Interclasse (CCI), coeficiente utilizado para mensurar a confiabilidade das medidas na comparação entre dois ou mais avaliadores^(10)^.

Foram ainda analisadas a interpretação do conteúdo escrito, bem como questões gramaticais e semânticas. Nesse processo, foram consideradas sugestões de acréscimo de alternativas de respostas ou de perguntas, bem como sugestões de adequação das questões que compunham o instrumento. Além disso, as reações não verbais, tais como expressões faciais, que sugeriram dúvida por parte dos juízes, em relação ao instrumento, foram analisadas^(11)^.

A análise dos dados obtidos foi realizada de forma qualitativa e quantitativa. De um ponto de vista qualitativo, as respostas foram analisadas a partir da descrição dos juízes acerca das suas impressões gerais sobre o instrumento, suas dúvidas durante a aplicação deste, sendo consideradas sugestões de mudança, acréscimo ou supressão de itens. Também, foram considerados: 1) o tempo utilizado para aplicação do instrumento; 2) a compreensão dos processos psicológicos e cognitivos referentes ao instrumento; 3) as diferenças de interpretação. De uma perspectiva quantitativa, as respostas foram avaliadas por meio das frequências relativas e absolutas. As interpretações individuais, o número absoluto e percentual dos entrevistados, que não entenderam as questões, do ponto de vista semântico, e as sugestões de aperfeiçoamento do instrumento foram consideradas e analisadas^(11)^. Após a análise das respostas, realizou-se as modificações pertinentes, que culminou na elaboração de uma nova versão do IMRRBH, direcionada à população idosa.

## RESULTADOS

Os profissionais participantes do estudo têm, em média, 34 anos, sendo a idade mínima de 25 e a máxima de 53 anos. O perfil dos profissionais participantes, está descrito na Tabela 1.

**Tabela 1:** Distribuição dos dados quanto ao perfil dos participantes do estudo

Variável	n	%
Idade		
De 25 a 39 anos	12	80,0
40 anos ou mais	3	20,0
Nível acadêmico		
Pós-graduação *lato sensu*	12	80,0
Mestrado	3	20,0
Área de atuação		
Apenas prática clínica	6	40,0
Prática clínica e docência	9	60,0
Tempo de prática clínica		
Entre 2 e 5 anos	5	33,3
Entre 5 e 10 anos	3	20,0
10 anos ou mais	7	46,7

Legenda: n = número

Fonte: As autoras

### Respostas verbais

O valor do IVC variou entre 0,93 e 1,0. O valor ideal para considerar um alto nível de concordância é 0,90^(10)^. Estes dados estão descritos na Tabela 2.

**Tabela 2:** Respostas Verbais

Questão	IVC	Profissionais consideraram a questão relevante ou muito relevante n (%)	Profissionais consideraram a questão claramente escrita (%)	Profissionais conservaram a escrita das questões sem quaisquer sugestões de mudanças (%)	Profissionais conservaram o binômio de resposta sim/não (%)	Profissionais conservaram as perguntas sem sugestões de acréscimos (%)
**1)** Paciente apresenta idade ≥ a 60 anos?	1	15 (100)	80	73,3	93,3	100
() sim () não						
**2)** Apresenta histórico de doença pregressa? (Neurológica, Respiratória, Esofágica, Gástrica, Cx de Cabeça e Pescoço)	1	15 (100)	86,6	80	93,3	86,6
() sim () não						
**3)** Paciente apresenta escala de Glasgow < 13?	1	15 (100)	73,3	66,6	100	93,3
() sim () não						
**4)** Necessitou de Intubação Orotraqueal?	1	15 (100)	73,3	60	86,6	86,6
() sim () não						
**5)** Tempo de Intubação Orotraqueal ≥ 24 h?	0,93	14 (93,3)	80	46,6	93,3	86,6
() sim () não						
**6)** Utiliza traqueostomia?	1	15 (100)	100	86,6	100	73,3
() sim () não						
**7)** Paciente apresenta sensação de dispneia?	1	15 (100)	100	86,6	86,6	66,6
() sim () não						
**8)** Paciente apresenta higiene oral inadequada?	0,93	14 (93,3)	73,3	53,3	93,3	60
() sim () não						
**9)** Paciente utiliza via alternativa de alimentação? (SNE, GTT, Jejunostomia)	1	15 (100)	100	86,6	86,6	73,3
() sim () não						
**10)** Paciente apresenta tosse/engasgo durante a alimentação ou com a saliva?	1	15 (100)	100	100	93,3	66,6
() sim () não						

Legenda: IVC = Índice de Validade de Conteúdo; SNE = Sonda Nasoenteral; GTT = Gastrostomia

Fonte: As autoras

Já o CCI variou entre 0,88 e 0,97, indicando um nível excelente de concordância^(10)^. Dessa forma, o valor elevado do CCI sugere que a variabilidade entre as respostas dos avaliadores foi baixa, indicando resultado positivo na análise de concordância. Os cálculos destes resultados estão descritos na Tabela 3.

**Tabela 3:** Coeficiente de Correlação Intraclasse - CCI

**ITEM**	**CCI**	Intervalo de confiança - 95%	
		Limite Inferior	Limite Superior
1	0,952	0,886	0,999
2	0,961	0,883	0,999
3	0,945	0,836	0,999
4	0,925	0,878	0,999
5	0,891	0,868	0,999
6	0,906	0,882	0,999
7	0,962	0,886	0,999
8	0,884	0,865	0,999
9	0,969	0,908	0,999
10	0,973	0,921	0,999
**Total**	**0,931**	**0,900**	**0,996**

Fonte: As autoras

### Respostas não verbais

Além do questionário respondido pelos profissionais participantes, foram verificadas respostas não verbais, em função de quatro gestos expressados por eles: hesitação, franzimento da testa, inclinação da cabeça e direcionamento da mão à boca. Os dados obtidos estão descritos na Tabela 4, a qual indica que houve, em cada item, pelo menos um tipo de gesto indicando uma resposta não verbal. Nos itens 3 e 8, observou-se hesitação mais frequente – em que, pelo menos três (20,0%) dos participantes, expressaram o referido gesto – além de franzimento da testa, inclinação da cabeça e direcionamento da mão à boca, no caso do item 8. Em contrapartida, observou-se que, no item 9, apenas um (6,7%) dos participantes expressou exclusivamente o gesto de franzir a testa, em resposta não verbal ao item.

**Tabela 4:** Distribuição de frequência das respostas não verbais por item

**ITENS**	**Hesitação**		**Franzir a testa**		**Inclinar a cabeça**		**Mão na boca**	
	n	%	n	%	n	%	n	%
1	2	13,3	1	6,7	-	-	-	-
2	1	6,7	2	13,3	-	-	1	6,7
**3**	**4**	**26,7**	**2**	**13,3**	**1**	**6,7**	**-**	**-**
4	2	13,3	2	13,3	1	6,7	-	-
5	2	13,3	-	-	1	6,7	-	-
6	1	6,7	-	-	1	6,7	-	-
7	2	13,3	-	-	-	-	1	6,7
**8**	**3**	**20,0**	**1**	**6,7**	**2**	**13,3**	**2**	**13,3**
**9**	**-**	**-**	**1**	**6,7**	**-**	**-**	**-**	**-**
10	1	6,7	-	-	-	-	1	6,7

Fonte: As autoras

Em relação às sugestões gerais do instrumento, na questão 2, um juiz referiu que necessitou explicar a pergunta para o paciente e outro juiz relatou necessidade de dar exemplos de doenças, às quais se referia. Já, na questão 3, um juiz sugeriu acrescentar a escala RASS – *Richmond AgitationSedationScale*, caso o instrumento pudesse ser adaptado, para ser utilizado em Unidades de Terapia Intensiva. Três juízes referiram que desconheciam a escala de coma de Glasgow, portanto, não tinham familiaridade para aplicar a questão. Ainda com relação a esta questão, um juiz referiu que a escala de coma de Glasgow é validada para avaliar pacientes que sofreram trauma, sugerindo que fosse usada uma escala mais adequada para rastreio de risco de broncoaspiração sem, contudo, indicar outra opção.

Na questão 4, um juiz referiu que necessitou explicar ao paciente o termo “Intubação Orotraqueal”. Na questão 7, um juiz sugeriu que o profissional aplicador pudesse descrever se percebeu sinais de desconforto respiratório durante o rastreio. Outro juiz referiu que necessitou explicar o significado da palavra “dispneia”. Por fim, na questão 10, foi sugerido, por um juiz, que o profissional aplicador pudesse descrever questões relacionadas à eficiência da tosse do paciente. Como sugestões gerais do instrumento, foram indicadas a inclusão da identificação do paciente, bem como a data da aplicação do instrumento de rastreio e o número do prontuário do paciente.

Após análise das sugestões dos juízes, foram realizadas modificações no instrumento, sendo considerados dados pertinentes para a facilitação da utilização do mesmo. Essas adequações estão dispostas no Quadro 1.

**Quadro 1:** Adequações realizadas após a análise das entrevistas e consenso entre os autores

*Questões da primeira versão do Instrumento Multiprofissional de Rastreio para Risco de Broncoaspiração em Ambiente Hospitalar.*	*Adequações para a segunda versão do Instrumento*
1. Paciente possui ≥60 anos?	Retirado formato de pergunta e realizado adequação das respostas desta questão. Substituição do símbolo por sua escrita por extenso, por se entender que causaria menos confusão para indivíduos pouco familiarizados com a simbologia.
2. Apresenta histórico de doença pregressa? (Neurológica, Respiratória, Esofágica, Gástrica, Cx de Cabeça e Pescoço)	Inclusão da palavra “atual”, contemplando assim não só o histórico de doenças prévias e modificação nas sugestões de doenças incluindo também “doenças de cabeça e pescoço”.
3. Paciente apresenta escala de Glasgow < 13?	Substituição do símbolo por sua escrita por extenso.
4. Necessitou de Intubação Orotraqueal?	Inclusão do termo “neste internamento? ”, por entender que este cenário impactaria na condição atual do paciente.
5. Tempo de Intubação Orotraqueal ≥ 24 h?	Substituição do símbolo por sua escrita por extenso. Inclusão do termo “neste internamento” e inclusão da opção de resposta “não aplicável”.
6. Utiliza traqueostomia?	Sem alteração
7. Paciente apresenta sensação de dispneia?	Inclusão da opção de resposta “não aplicável”, considerando que a resposta a esta pergunta somente o paciente possa responder e alguns pacientes não consigam devido seu estado clínico.
8. Paciente apresenta higiene oral inadequada?	Substituição do termo inadequada, para adequada.
9. Paciente utiliza via alternativa de alimentação? (SNE, GTT, Jejunostomia)	Acréscimo do termo “como única via”, já que alguns pacientes necessitam desta via somente como complemento nutricional. Inclusão da via alternativa parenteral (NPT), por ser uma via de alimentação presente no ambiente hospitalar.
10. Paciente apresenta tosse/engasgo durante a alimentação ou com a saliva?	Inclusão da opção de resposta “não aplicável”, considerando que a resposta a esta pergunta somente o paciente possa responder e alguns pacientes não consigam devido seu estado clínico atual ou doenças de base.

Legenda: SNE = Sonda Nasoenteral; GTT = Gastrostomia; NPT = Nutrição Parenteral

Fonte: As autoras

Na sequência, no Quadro 2, estão dispostas as questões da primeira versão do instrumento, as sugestões dos juízes e o que foi considerado para a segunda versão do instrumento.

**Quadro 2:** Dados comparativos entre a primeira e segunda versão do instrumento

**Itens que compunham a primeira versão do Instrumento**	**Sugestões de modificação**	**Itens considerados para a segunda versão do instrumento**
1)Paciente apresenta idade ≥ a 60 anos? *Resposta:* () Não () Sim	*Pergunta: a)* Idade: b) Paciente apresenta idade maior ou igual a 60 anos? *Resposta:* () 60-74 anos () mais de 75 anos	1) Idade: () entre 60 e 74 anos () mais de 75 anos
2) Apresenta histórico de doença pregressa? (Neurológica, Respiratória, Esofágica, Gástrica, Cx de Cabeça e Pescoço) *Resposta:* () Não () Sim	*Pergunta: a)* Histórico de doença pregressa: (Neurológica, Respiratória, Esofágica, Gástrica, Cx de Cabeça e Pescoço). b) Apresenta histórico de doença atual ou pregressa? (Neurológica, Respiratória, Esofágica, Gástrica, Cx ou neoplasia de Cabeça e Pescoço); c) Apresenta histórico de doença pregressa? (Acrescentar: AVC, Parkinson, demência *Resposta: a)* () presente () ausente b)Quais comorbidades?	2) Apresenta histórico de doença atual ou pregressa? (De ordem neurológica, gástrica, respiratória ou doença de cabeça e pescoço) *Resposta:* () Não () Sim
3) Paciente apresenta escala de Glasgow < 13? *Resposta:* () Não () Sim	*Pergunta: a)* Paciente apresenta escala de Glasgow maior que 13? b) Glasgow menor que 13: c) Paciente apresenta escala de Glasgow menor que 13? d) Este é o estado de vigília basal do paciente?	3) Paciente apresenta escala de Glasgow menor que 13? *Resposta:* () Não () Sim
4) Necessitou de Intubação Orotraqueal? *Resposta:* () Não () Sim	*Pergunta: a)* Necessitou de Intubação Orotraqueal neste internamento? b) Intubação Orotraqueal: c) Quantos dias? d) Quanto tempo? d) Neste internamento? e) Ficou internado na UTI?	4) Necessitou de Intubação Orotraqueal neste internamento? *Resposta:* () Não () Sim
5) Tempo de Intubação Orotraqueal ≥ 24 h? *Resposta:* () Não () Sim	*Pergunta: a)* Tempo de Intubação Orotraqueal maior ou igual a 24 h? b) Intubação Orotraqueal ≥ 24 h: c) Tempo de Intubação Orotraqueal ≥ 24 h, d) neste internamento? e) Quantos dias? f) Causa da IOT?*Resposta:* () Não aplicável	5) Tempo de Intubação Orotraqueal maior ou igual a 24 h, neste internamento? *Resposta:* () Não () Não aplicável () Sim
6) Utiliza traqueostomia? *Resposta:* () Não () Sim	*Pergunta: a)* Traqueostomia: b) Utiliza traqueostomia neste internamento? c) Apresentou falha de extubação? d) Há quanto tempo utiliza? e) Desde este internamento? f) Já utilizou? *Resposta:* () presente () ausente	6) Utiliza traqueostomia? *Resposta:* () Não () Sim
7) Paciente apresenta sensação de dispneia? *Resposta:* () Não () Sim	*Pergunta: a)* Dispneia: b) Paciente apresenta falta de ar? c) Faz uso de suporte de O2? d) Em que momentos do dia? e) Em que momentos é mais intensa? f) Com qual frequência? g) Este foi o motivo do internamento? *Resposta:* () não aplicável	7) Paciente apresenta sensação de “falta de ar”? *Resposta:* () Não () Não aplicável () Sim
8) Paciente apresenta higiene oral inadequada? *Resposta:* () Não () Sim	*Pergunta: a)* Paciente apresenta higiene oral adequada? b) Higiene oral: c) Paciente apresenta higiene oral deficitária? d) Utiliza prótese dentária? e) Faz sua higiene oral sozinho? f) Com qual frequência realiza sua higiene oral? g) Porque a higiene oral é ruim? *Resposta: a)* () Parcialmente adequada	8) Paciente apresenta higiene oral adequada? *Resposta:* () Sim () Não
9) Paciente utiliza via alternativa de alimentação? (SNE, GTT, Jejunostomia) *Resposta:* () Não () Sim	*Pergunta: a)* Via alternativa de alimentação: b) Paciente utiliza via alternativa de alimentação como única via? (SNE, GTT, Jejunostomia) c) Paciente utiliza via alternativa de alimentação? (SNE, GTT, Jejunostomia, NPT) d) Há quanto tempo utiliza via alternativa de alimentação? e) Já utilizou anteriormente? f) Qual o motivo que está utilizando o dispositivo?	9) Paciente utiliza sonda de alimentação como única via? (SNE, GTT, Jejunostomia, NPT). *Resposta:* () Não () Sim
10) Paciente apresenta tosse/engasgo durante a alimentação ou com a saliva? *Resposta:* () Não () Sim	*Pergunta: a)* Posicionamento no leito: _______ b) Qual a consistência que o paciente come? c) Com quais alimentos? d) Há quanto tempo apresenta estes episódios? e) Quando iniciou estes sintomas? f) Com que frequência? g) Qual a causa da tosse/engasgo? *Resposta: a)* () não aplicável	10) Paciente apresenta tosse/engasgo durante a alimentação ou com a saliva? () Não () Não aplicável () Sim

Legenda: SNE = Sonda Nasoenteral; GTT = Gastrostomia; NPT = Nutrição Parenteral

Fonte: As autoras

Após análise dos resultados obtidos durante a entrevista, foi elaborada a segunda versão do IMRRBAH à população Idosa, na qual foi incluído um guia de instruções por escrito, a fim de facilitar a sua aplicação, pelos profissionais.

## DISCUSSÃO

A proposta desta pesquisa foi aprimorar o IMRRBAH à população idosa, o qual foi desenvolvido para a aplicação por profissionais da saúde envolvidos na assistência direta ao paciente. Para isto, optou-se por encontrar evidência de validação baseada nos processos de resposta, através de entrevista dirigida e análise dos processos psicológicos envolvidos.

Em relação ao perfil dos juízes, observa-se que todos tinham alguma especialização e predominavam profissionais que, além da atuação clínica, a qual a maioria exercia há mais de 10 anos, também exerciam docência. Neste sentido, a literatura aponta que as habilidades desenvolvidas por profissionais especializados têm importante função para a permanente constituição dos profissionais da saúde, sendo capaz de modificar a prática destes através de novos modelos assistenciais, contribuindo de forma efetiva com instituições em que estão inseridos e beneficiando a população atendida^(12)^.

Para a análise quantitativa das respostas verbais, foi utilizado o IVC, por ser um método bastante utilizado na área da saúde para mensurar a concordância dos juízes em relação a cada item que compõe um determinado instrumento individualmente através da escala Likert^(13)^.

De acordo com a maior parte dos juízes, a escrita dos itens estava clara. Além disso, a maioria afirmou que manteria a forma de apresentação das perguntas e das respostas, fato que se apresenta em concordância com a literatura científica, a qual refere que instrumentos validados devem ser compostos por itens coerentes e relevantes sobre o tema a que se propõe avaliar, evitando dúvidas por parte do aplicador^(14)^.

Em relação às respostas não verbais obtidas, destacam-se as encontradas no item 8 do instrumento, referente a higiene oral do paciente, por ter sido a questão que provocou maior estranhamento por parte dos juízes., Por isso a sugestão de alteração do termo “inadequada” para “adequada”, foi considerada.

As demais sugestões dos juízes e consideradas pertinentes foram eleitas para compor a nova versão do instrumento, além do guia de instrução para sua aplicação.

A busca por outras indicações de validade é necessária, especialmente no que concerne à validação dos escores atribuídos nas etapas anteriores, a fim de obter dados mais consistentes e concretos ao rastreio sobre quando acionar o fonoaudiólogo para realizar avaliação clínica da deglutição. Em busca recente, verificou-se que o dispositivo, objeto deste estudo, ainda é o único na literatura científica que se propõe a rastrear o risco de broncoaspiração, fato que demonstra a relevância da continuidade desta pesquisa. Os materiais encontrados na literatura procuram rastrear o risco de disfagia^(15,16)^, que sim, é um preditor de risco para broncoaspiração, no entanto não deve ser o único a ser considerado. Outros estudos ainda apontam para a importância de prevenir a broncoaspiração e sugerem protocolos de prevenção para a condição^(17,18).^

Sobre as limitações do estudo, cabe ressaltar que o mesmo foi realizado em um hospital referência no atendimento aos idosos, o que restringiu a população da amostra.

## CONCLUSÃO

Os resultados obtidos na terceira etapa desta pesquisa demonstraram, por meio de dados dos processos psicológicos e cognitivos dos juízes, que a validade do IMRRBAH junto à população idosa, baseada nos processos de respostas, foi alcançada.
